# Supplementary material for: Computer-Based Cognitive Training in Children with Primary Brain Tumours: A Systematic Review
Source: Cancers (Basel). 2022 Aug 11;14(16):3879. doi: 10.3390/cancers14163879 (PMC9405613; doi:10.3390/cancers14163879)
Supplement: Supplementary file 1 [file cancers-14-03879-s001.zip › cancers-1801178-supplementary.pdf]

cancers

MDPI

Digital Interventions Employed in the Included Studies

1. Cogmed [1]: Cogmed is a personalized, digital therapeutic, designed to improve the brain systems responsible for attention and working memory. Working memory is closely related to the control of attention, and uses, in part, the same regions of the brain. Cogmed training is specifically developed to target these brain regions.
   The program consists of a range of finely tuned exercises, which adapt dynamically to the user's capacity and progression. This keeps the user in the zone of optimal effort, which is necessary to gain the desired results.
   The training is carried out on a computer, smartphone or tablet, and consists of a series of short exercises that forces the person training to push the limits of their working memory. Performing the training can be fun, and at times frustratingly challenging.
   The exercises are carefully designed to challenge the attention and working memory of the person training. As the trainee improves at an exercise, the level of difficulty increases. The program is adaptive to ensure the training and remainsat the optimal level of effort.

2. Fast ForWord [2]: Fast ForWord is an evidence-based, adaptive reading and language program that delivers 1–2 years of gain in 40–60 h of use for any struggling learner.
   Unlike most reading programs and solutions that work around the underlying weaknesses that cause children's reading difficulty, Fast ForWord targets the foundational cognitive skills required for reading and learning: Working memory, attention, auditory processing, and sequencing. Moreover, a remote-flexible solution, the Fast ForWord software provides robust and actionable reports of demonstrable reading skill gains, as measured in grade level reading.
   Fun characters, animated rewards, real-time progress meters, and other features engage patients as they work on gamified exercises that adapt to patients' performance. Motivated patients who work on this personalized and adaptive program make rapid progress, no matter their starting point. Age-appropriate content, adaptive exercises, and levelled reading selections dynamically adjust to reading skill attainment and patient progress. Exercises are designed to promote confidence while providing real-time and personalized corrective feedback.

3. Captain's Log [3]: The fifty multi-level programs in the Captain's Log system are organized into three training sets: (i) Attention Skills Training Set, (ii) Problem Solving and Memory Skill Training Set, (iii) Working Memory Training Set.

   Attention Skills Training Set

   The Attention Skills Training Set is comprised of three modules of Captain's Log: *Attention Skills Developmental Module, Visual Motor Skills Module, and Attention Skills the Next Generation*.

   Attention Skills Developmental Module

   These eight programs begin with simple exercises for patients with head injuries or developmental disabilities.

   Visual Motor Skills Module

   These seven popular programs are played as video games, but work to train hand-eye coordination, visual processing, and fine motor control.

   Attention Skills the Next Generation Module

   Using this set further enhances higher level attention skills and memory. These three challenging programs continue to build on the skills addressed in the Attention Skills Developmental module.

   Problem Solving and Memory Skills Training Set

   The Problem Solving and Memory Skills Training Set is comprised of the *Conceptual Memory Skills, Logic Skills, and Numeric Concepts with Memory Skills modules of Captain's log*.

<u>*Conceptual Memory Skills Module*</u>

These seven programs train the impulse control, working memory, processing speed, and logical reasoning.

<u>*Logic Skills Module*</u>

This module features five engaging programs to help improve executive functioning. Patients learn deductive reasoning, understanding of relationships between numbers and objects, and perception of how parts fit into a whole.

<u>*Numeric Concepts with Memory Skills Module*</u>

The challenging games in these five higher level programs train working, immediate, and short-term visual memory, deductive and inductive reasoning, visual discrimination, categorization, and sequencing.

Working Memory Training Set

Working memory training improves patients' ability to learn and remember. This training set provides 625 h of working memory training.

The Working Memory Training Set is comprised of three Captain's Log modules*: Real Life Working Memory, Working Memory Skills, and Auditory Working Memory*.

<u>*Real Life Working Memory Module*</u>

These five programs are both practical and entertaining. Patients learn to put their working memory to work for them in real life situations.

<u>*Working Memory Skills Module*</u>

The five programs in this module train memory for names and faces, sequential pattern recognition, list recall, and more (skills that are vital for success in school and in life).

<u>*Auditory Working Memory Module*</u>

These five brain training programs focus on working memory and auditory attention. Patients with attention deficits will train both their working memory and listening skills.

4. <u>Wii-Fit</u> [4]: Wii Fit is a health and fitness game that helps your body coordination. Exercise routines can be performed based on which specific areas of fitness the participant wants to focus on. Thanks to the Wii Balance Board and motion-sensing power of Wii, Wii Fit makes it fun to experience exercise at home. As well as a huge variety of exercises, players can enjoy active gaming via 15 Training Plus games designed to offer a fun home workout for both mind and body: From taking part in a snowball fight to hitting the driving range, while keeping it very entertaining. The games train crucial systems associated with the movement: Body coordination, hand-eye coordination, and fine motor control.

References

1. Available online: https://www.cogmed.com (accessed on 12 January 2022).
2. Available online: https://www.scilearn.com/program (accessed on 12 January 2022).
3. Available online: https://www.braintrain.com/captains-log-for-educators/ (accessed on 12 January 2022).
4. Available online: https://www.nintendo.com (accessed on 12 January 2022).
